# Examining Predictors of Nonsuicidal Self-Injury Reveals Across the First Year of University

**DOI:** 10.1177/21676968261474577

**Published:** 2026-07-30

**Authors:** Brooke C. T. Farrell, Chloe A. Hamza

**Affiliations:** 1 Department of Applied Psychology & Human Development, Ontario Institute for Studies in Education, 113749 University of Toronto, Toronto, ON, Canada

**Keywords:** nonsuicidal self-injury, post-secondary, emerging adults, disclosure, longitudinal, at-risk populations, quantitative methods, self harm, social support

## Abstract

The transition to university represents a novel social context for nonsuicidal self-injury (NSSI) experiences to be shared among emerging adults. However, longitudinal work on NSSI reveal experiences among young adults is scarce, precluding an understanding of factors that promote reveals and help-seeking. To address these gaps, predictors of NSSI reveals among 592 university students (*M*_age_ = 18.11, 72% female) with lifetime histories of NSSI were examined. Participants completed an online survey three times (3 months apart) over their first year of university. It was found that 50% of students reported an NSSI reveal at some point across their first year of university. Binary logistic regression analyses showed that students reporting greater NSSI lifetime severity at baseline were more likely to later report a voluntary disclosure, while students reporting greater depressive symptoms at baseline were more likely to later report an involuntary discovery. Findings inform NSSI response efforts on post-secondary campuses.

## Introduction

The transition to university marks a sensitive developmental period for emerging adults (ages 18–25 years), characterised by significant life changes and increased academic demands (American College Health Association, 2020). As a result, many first-year university students experience heightened stress (Garett et al., 2017) and may engage in various coping strategies throughout this challenging time (Freire et al., 2020). One prevalent form of risky coping is nonsuicidal self-injury (NSSI), defined as the purposeful and direct damage of one’s body tissue (e.g., cutting, burning), engaged in without suicidal intent, and in ways that are not socially sanctioned (International Society for the Study of Self-Injury, 2018). Although NSSI often has its onset in adolescence (Swannell et al., 2014), emerging adulthood is also a period of increased risk, with as many as 18% of post-secondary students indicating lifetime engagement and 8% indicating recent engagement (Kiekens et al., 2023). This is concerning as NSSI is associated with academic underperformance (Kiekens et al., 2016), interpersonal difficulties (Turner et al., 2016), and other mental health concerns among students (e.g., depression, anxiety, suicidality) (Kiekens et al., 2023).

Findings indicate that self-disclosure (i.e., the sharing of a concealable stigmatized identity) of NSSI can facilitate social connectedness, intimacy, and formal support seeking (Gayfer et al., 2020; Rosenrot & Lewis, 2020). In this way, NSSI disclosures may represent an important experience that may foster NSSI recovery (Hasking et al., 2015, 2024; Lewis & Hasking, 2021). However, it is important to recognize that NSSI is a highly stigmatized behaviour (Burke et al., 2019; Lewis & Seko, 2016), and having others come to know of one’s self-injury can represent a stressful experience for some students that may lead to negative psychological and social consequences (e.g., feelings of shame and guilt, rejection from one’s family or peers) (Buser et al., 2014; Rosenrot & Lewis, 2020). As such, some students may choose to conceal this behaviour (Armiento et al., 2014; Rosenrot & Lewis, 2020), limiting access to professional help and emotional support from loved ones. Nevertheless, recent findings indicate that many post-secondary students do have others come to know of their NSSI in some way (Ammerman & McCloskey, 2021; Mirichlis et al., 2022; Simone & Hamza, 2023). These reveal experiences may be particularly relevant to help-seeking and recovery in the first year of university, as this may represent a novel social context for NSSI experiences to be shared. Indeed, emerging evidence suggests that emerging adults may be more likely to disclose NSSI than adolescents, which may stem from new social opportunities for disclosure (Kim et al., 2025). Further, the transition to university, specifically, represents a period of vulnerability (Cage et al., 2021), as students navigate new personal, academic, and social stressors (e.g., living away from home for the first time, adjusting to new academic demands, and developing new relationships) (Linden & Stuart, 2020), as well as increased risk for NSSI engagement (Farrell et al., 2024; Wester et al., 2018). As such, elucidating the factors that may promote NSSI reveals is critical to understanding how to foster help-seeking among post-secondary students.

### Types of NSSI Reveals

Although recent research efforts have begun to elucidate voluntary NSSI disclosure experiences, existing work has overwhelmingly conflated the different ways in which NSSI may be revealed to others. Specifically, a significant number of studies have been unclear on whether “disclosures” were actually intentionally self-disclosed or unintentionally discovered. Studies often ask participants how many people *have knowledge* of their NSSI without specifying the way in which this information was obtained (see Simone & Hamza, 2020 for a review). As such, current understanding of involuntary discoveries of NSSI (e.g., seeing scarring) and indirect disclosures of NSSI (e.g., a confidant tells someone else without consent) remains limited. Further, existing research on voluntary disclosures may be confounded by the inclusion of these other reveal experiences. Indeed, involuntary discoveries and indirect disclosures may be characterized by reduced autonomy and preparedness (Lewis & Hasking, 2023; Pugh et al., 2023), making them notably distinct from the voluntary decision to disclose one’s NSSI. The little research that has distinguished voluntary and involuntary reveal experiences has found that involuntary discoveries of NSSI do occur (Ammerman et al., 2021; Turner et al., 2016), but research is needed to elucidate the antecedents and outcomes of these unique experiences. Taken together, disentangling these experiences is critical to identifying the different ways in which NSSI is revealed in a post-secondary context, what promotes these different experiences, and how they may differ in their impact on individuals’ well-being, recovery, and help-seeking.

### Potential Predictors of Different NSSI Reveals

#### Difficulties With Emotion Regulation

Recent findings highlight the seeking of emotional support as a common motivation for undergraduate students to disclose their NSSI (Ammerman et al., 2021; Simone et al., 2023). This is in line with the considerable evidence for the relation between emotion dysregulation and a higher risk for NSSI (Wolff et al., 2019), with theories (Nock’s, 2010) highlighting the role of NSSI as a form of emotional coping in the context of stress (e.g., the transition to university). Emotion dysregulation has further been identified as a transdiagnostic risk factor for psychological disorders (Aldao et al., 2016) and may serve as an indicator of mental health concerns more broadly (Aldao et al., 2010). It is possible then, that students exhibiting greater difficulties with emotion regulation may experience more distress, leading them to disclose their NSSI in attempts to seek formal (e.g., through counselling) or informal (e.g., confiding in a friend) emotional support. Furthermore, it is possible that students with greater emotion dysregulation may be more likely to then experience indirect disclosures due to increased concern from confidants regarding their higher levels of distress.

#### Depressive Symptoms

Existing work also suggests that depressive symptoms may influence NSSI reveal experiences. Ammerman and colleages (2021) found that lower levels of depression were associated with a higher likelihood of voluntary disclosures, suggesting that key symptoms of depression (e.g., low motivation, fatigue, and social withdrawal) may represent barriers to intentional self-disclosure. However, the role of depressive symptoms in involuntary and indirect NSSI reveals remains underexplored. It is possible that increased depression may lead to involuntary discoveries if individuals are less motivated to conceal visible signs of self-injury. Indirect disclosure may also occur when confidants, concerned by signs of depressive distress, share information with others in attempts to secure help for the individual. Additionally, the impact of depression may extend beyond initiating disclosures to how reveals are received. For example, Park and Ammerman (2020) found that negative responses to NSSI disclosure exacerbated depressive symptoms, which subsequently increased suicide risk. Other research indicates that individuals who disclose NSSI report more suicidal thoughts than those who do not disclose (Long et al., 2015). Further investigation is required to elucidate how depressive symptoms may influence the dynamics of other types of reveals, as students with increased depressive symptoms may be uniquely sensitive to reveal responses and require formal help to navigate these experiences.

#### NSSI Severity

The severity of NSSI itself may play a role in NSSI reveals. NSSI versatility (i.e., the number of distinct methods of NSSI) is a well-established indicator of NSSI severity due to its association with suicidal behaviour (Anestis et al., 2015; Turner et al., 2013). A recent longitudinal study revealed that post-secondary students who reported more severe NSSI behaviors were more likely to disclose their NSSI over time (Simone & Hamza, 2021), and other researchers have found that individuals who disclose NSSI typically report greater NSSI severity as well (Armiento et al., 2014; Hasking et al., 2015). Understanding how NSSI severity plays a role in other reveal experiences is an important next step in this area of work, as increased visible wounds and scarring may lead to involuntary discoveries, and NSSI severity may signal greater distress resulting in indirect disclosures from concerned confidants.

#### Loneliness

In recent years, loneliness has become an increasing concern underlying mental health challenges in university students (Fagan et al., 2023; Lasgaard et al., 2016). The transition to post-secondary requires many students to re-locate, resulting in a new social landscape and community of peers. For some students, this may represent a positive experience in which they feel a sense of belonging (Maymon & Hall, 2021) and could provide an opportunity for the sharing of NSSI experiences. Access to resources on campus could further provide new means for help-seeking and disclosure to formal supports (Simone & Hamza, 2021). However, for others, this new environment may lead to feelings of isolation and remove important social supports from students’ previous contexts (i.e., family and friends who are no longer in close proximity) (McLean et al., 2023). As such, loneliness may represent a salient barrier to sharing NSSI experiences in the unique context of the transition to university. This is in line with research that has found that voluntary disclosures are often associated with greater social support (Ammerman et al., 2021; Mirichlis et al., 2022) and greater friendship quality (Armiento et al., 2014). It is possible that loneliness may also serve as a barrier to involuntary discoveries and indirect disclosures due to reduced social contact and isolation.

#### Self-Efficacy to Resist NSSI Urges

Disclosure may play a significant role in NSSI recovery (Lewis & Hasking, 2021), both as a tool to seek support from loved ones and as a gateway to more formal help-seeking (Hasking et al., 2015). Recovery may be specifically relevant to disclosures in the post-secondary context, as Armiento and colleages (2014) found that undergraduate students who disclosed NSSI had a greater desire to stop self-injuring. Recent person-centered work on NSSI recovery highlights that it is a nonlinear process (i.e., progress can occur despite setbacks) and is multifaceted, involving more than cessation (Lewis & Hasking, 2023). Importantly, normalizing thoughts and urges of self-injury, and building self-efficacy to resist urges, are salient aspects of recovery (Lewis & Hasking, 2021). Some work has demonstrated that the self-efficacy that develops in the context of resisting urges appears to translate to other areas of individuals’ lives as well (Farrell et al., 2023). It is possible that this increased confidence could then extend to individuals’ perceived ability to navigate disclosures and discoveries of their NSSI. Further exploration may provide important insight to the intersection of NSSI reveals and recovery.

#### Healthy Coping

Coping styles may represent a salient predictor of NSSI reveals as well. Hasking and colleagues (2015) found that adolescents who disclosed their NSSI reported less problem focused coping, and more non-productive coping than those who did not disclose. However, little is known about how unsuccessful attempts at healthy coping may lead to disclosure. It is possible that students engaging in NSSI may attempt to cope using a variety of both healthy and unhealthy strategies (i.e., coping flexibility) which have little effect due to high levels of distress (Ewing & Hamza, 2024), leading to disclosure in attempts to seek help. In contrast, individuals who report engaging in more healthy coping may be further along in their recovery and feel more confident in their ability to navigate disclosures or discoveries of NSSI (e.g., no longer concealing scarring).

### Lack of Longitudinal Research

Despite preliminary findings on potential predictors of NSSI reveals, much of this work has been limited by a lack of longitudinal work, precluding an understanding of the factors that promote different NSSI reveals over time. Exploring reveal experiences over time can provide a more nuanced understanding of the reveal process and developmental patterns of NSSI disclosure/discovery. Indeed, existing research has found that disclosure appears to be an ongoing process that occurs over time (e.g., a student starts by telling a peer, which leads to them feeling comfortable to tell a parent, and then to seek formal help) rather than a single event (Hasking et al., 2024; Simone & Hamza, 2021). However, identifying factors that temporally precede reveals is necessary to understand the processes through which disclosures, and potential help-seeking, may occur. Given that the transition to university may provide a unique and new social context for reveals to occur, understanding the factors that precipitate these experiences represents an important line of inquiry.

### Present Study

Although the post-secondary years are a period of increased vulnerability for NSSI (Kiekens et al., 2023), and may represent a context for new NSSI reveals to occur, limited work has focused on NSSI reveals among post-secondary students. In particular, there is a paucity of longitudinal work precluding an understanding of what may promote NSSI reveal experiences in the post-secondary context over time. Furthermore, existing work has often conflated the different ways in which NSSI reveals may occur. To address these gaps, the present study aimed to examine multiple relevant predictors of different NSSI reveal experiences (i.e., voluntary disclosure, involuntary discoveries, and indirect disclosure) across the first year of university using a longitudinal design. Based on prior literature, it was hypothesized that greater difficulties with emotion regulation, lower depressive symptoms, greater NSSI severity, lower perceived loneliness, greater self-efficacy to resist urges, and more healthy coping strategies at baseline would predict later voluntary disclosures. In the present study, it was also examined whether these factors at baseline would predict later involuntary discoveries and indirect disclosures across the first year of university; however, no formal hypotheses were made given the exploratory nature of this work.

## Methods

### Participants

The present sample consisted of 592 emerging adults (*M*age = 18.11, *SD* = 0.88, 72% female, 21% male, 7% gender diverse) who reported a lifetime history of NSSI and were currently enrolled in their first year of university at a large post-secondary institution in Toronto, Canada. This sample was drawn from a larger longitudinal study (N = 1214), in which participants completed an online survey three times (3 months apart) over their first year of university during the 2022 – 2023 academic year. Participants were eligible if they were between 18 and 25 years of age, enrolled in their first year of an undergraduate degree at the University of Toronto, and lived in the area surrounding the university.

### Procedure

Participants were invited to partake in a larger longitudinal study regarding the experiences of first year university students. Multiple techniques were used to recruit students including printed flyers on campus and in residences, email listservs (e.g., student groups or clubs), social media, and in-person class announcements. Students interested in participating were instructed to contact the lab via phone or email to be screened for eligibility based on the above criteria. Eligible students were assigned a unique participant identification number and were provided with a link to the Qualtrics survey platform to complete the survey online. A survey link was sent via email for participants to complete the same survey at Times 2 and 3. Participants who completed the survey received $15 for completing the baseline survey, $20 for completing Time 2, and $25 for completing Time 3. This study was approved by the university’s research ethics board, and active informed consent was obtained from all participants at each of the three time points that they completed the survey. A list of resources was provided to all participants at the end of each survey.

### Measures – Baseline Assessment

#### Demographic Questionnaire

At baseline survey completion, participants were asked to provide basic demographic information, including their age, ethnicity (options included East Asian, White, South Asian, Arab/West Asian, Black, Hispanic, Filipino, South East Asian, West Indian, Aboriginal, and a free-response option to specify their ethnicity), and gender (options included man, women, transgender, non-binary, unsure, and a free-response option to specify their gender).

#### NSSI Behaviour, Functions, and Severity

Participants completed the Inventory of Statements about Self-Injury (ISAS, Klonsky & Glenn, 2009) at each time point to determine whether they had engaged in seven direct forms of NSSI (i.e., cutting, biting, burning, carving, severe scratching, banging/hitting self, and rubbing skin). Participants were asked to indicate their frequency of these NSSI behaviours within the past three months. In the present study, the summed number of different methods a given participants used to engage in NSSI (e.g., cutting, burning) was employed as an indicator of NSSI severity. Participants further reported on 13 different functions of their NSSI (i.e., affect regulation, interpersonal boundaries, self-punishment, self-care, anti-dissociation/feeling-generation, anti-suicide, sensation-seeking, peer-bonding, interpersonal influence, toughness, marking distress, revenge, and autonomy). Each function scale was comprised of three items, with each item ranging from scores of 0 (not relevant) to 2 (very relevant). As such, scores range from 0 to 6 for each of the 13 functions. In the present study, Cronbach’s alphas for NSSI behaviour frequency were low, but acceptable for each of the three time points (α = 0.56, α = 0.71, α = 0.72, respectively), consistent with prior research using community-based samples (Glenn & Klonsky, 2011). Given that fewer methods of NSSI are often reported amongst community-based samples (Xiao et al., 2022), it is not surprising that Cronbach’s values are on the lower side. The internal consistency of the ISAS Functions for the current study was good for each of the three time points (α = 0.83, α = 0.83, α = 0.80, respectively).

#### NSSI Reveal Experiences

Participants completed a scale adapted from Rosenrot and Lewis (2020) to indicate whether they had experienced an NSSI reveal in their lifetime (i.e., does anyone know about your NSSI?), as well as if they had experienced a reveal in the past 3 months at baseline (i.e., has anyone come to know that you engage in NSSI in the past three months?). If participants reported a reveal, they were asked which source knew about their NSSI (e.g., mother, friend, partner) and how they came to know about their NSSI. Specifically, participants indicated whether they had experienced: (1) a voluntary disclosure (e.g., a student shared their experiences with a peer), (2) an involuntary discovery (e.g., a peer saw their scars and asked them about their NSSI), and/or an indirect disclosure (e.g., a friend told a parent about their NSSI).

#### Difficulties With Emotion Regulation

Participants completed the Difficulties with Emotion Regulation Scale – Short Form (DERS-SF, Kaufman et al., 2016). This 18-item measure contains statements that indicate possible responses to negative affective states and general statements regarding awareness of affective states. Participants indicated on a scale from 1 (almost never, 0–10 %) to 5 (almost always, 91–100 %) how often they experienced the affective states in the provided statements (e.g., “When I’m upset, I become embarrassed for feeling that way” and “I pay attention to how I feel.),” with higher scores indicating greater difficulties with emotion regulation. This short form version of the scale has similar psychometric properties to the original measure, and the scale has shown good internal consistency, concurrent validity, and factorial validity for post-secondary students (Hallion et al., 2018; Kaufman et al., 2016). The internal consistency of the DERS-SF for the current study was excellent for each of the three time points (α = 0.90, α = 0.90, α = 0.90, respectively).

#### Depressive Symptoms

Participants completed the Center for Epidemiologic Studies Depression Scale – Revised (CESD-R, Eaton et al., 2004) at each time point. This 18-item scale is used to measure depression symptoms based on the American Psychiatric Association Diagnostic and Statistical Manual, fifth edition (DSM-5) and has been validated for use in the general population (Van Dam & Earleywine, 2011). Participants rated how frequently they experienced each statement within the “past week or so” on from 0 (“not at all or less than one day”) to 4 (“nearly every day for 2 weeks”). Examples of statements included, “Nothing made me happy” and “I felt like a bad person.” This measure has been validated for use in the general population (Irwin et al., 1999). The internal consistency of the CESD-R for the current study was excellent for each of the three time points (α = 0.93, α = 0.94, α = 0.94, respectively).

#### Loneliness

Participants completed the UCLA Loneliness Scale – Short Form (ULC-8-SF, Hays & DiMatteo, 1987). This 8-item measure contains statements that indicate levels of social isolation and loneliness (e.g., “I lack companionship”) and uses a 4-point scale ranging from 1 (I never feel this way) to 4 (I often feel this way), where higher scores suggest a higher degree of loneliness. This scale has demonstrated good reliability and validity (Xu et al., 2018). The internal consistency of the ULS-8-SF for the current study was good for each of the three time points (α = 0.84, α = 0.84, α = 0.85, respectively).

#### Self-Efficacy to Resist NSSI Urges

Participates completed an adapted version of the Self-Efficacy to Avoid Suicidal Action Scale (SEASA, Hays & DiMatteo, 1987) to assess perceived ability to resist NSSI urges. Participants rated six items on a scale of 1 (very uncertain) to 10 (very certain) about whether they believed they could resist engaging in NSSI in the next two weeks (e.g., “How certain are you that you will not self-injure in the next two weeks?”). The adapted version of this measure has demonstrated good internal consistency (Dawkins et al., 2021; Kiekens et al., 2020). The internal consistency of this scale for the current study was excellent for each of the three time points (α = 0.90, α = 0.90, α = 0.90, respectively).

#### Healthy Coping

Participants completed the Coping Index (CI, Stallman, 2017). This 21-item measure of healthy coping is based on the Health Theory of Coping (Stallman, 2020). Participants rated the extent to which they use all 20 strategies to feel better when they were anxious, stressed, or distressed on a scale of 0 (I don’t do this at all) to 3 (I do this most of the time), as well as one “Other” option where they could specify a strategy. A sum of the unhealthy coping strategies was subtracted from a sum of the healthy coping strategies to provide an overall measure of healthy coping. In the present study, three additional items were added to the healthy coping subscale, including exercising, running, and going to the gym. Furthermore, items including NSSI, thoughts of self-injury, and talking to a professional/counselor were removed from the scale to reduce confounding factors in subsequent analyses. The Coping Index has good test-retest reliability and internal consistency among university populations (Stallman, 2019; Stallman et al., 2022). The internal consistency of the healthy subscale of the Coping Index for the current study was moderate for each of the three time points (α = 0.66, α = 0.69, α = 0.71, respectively). The internal consistency of the unhealthy subscale of the Coping Index for the current study was moderate for each of the three time points (α = 0.60, α = 0.65, α = 0.66, respectively).

### Measures – Follow-Up Assessments

#### NSSI Behaviour, Functions, and Severity

Participants completed the adapted version of the ISAS (Klonsky & Glenn, 2009) again at Times 2 and 3, reporting on their recent (past 3 months) NSSI engagement.

#### NSSI Reveal Experiences

Participants completed a scale adapted from Rosenrot and Lewis (2020) again at Times 2 and 3, where they reported on their recent (past 3 months) reveal experiences (i.e., voluntary disclosures, involuntary discoveries, and indirect disclosures) to different sources.

### Data Analysis

Descriptive statistics were conducted to determine the prevalence of recent NSSI reveal experiences (i.e., voluntary disclosures, involuntary discoveries, and indirect disclosures) among participants (N = 592) across their first year of university (i.e., at baseline, Time 2, and Time 3). Binary logistic regression models were used to predict new types of reveals at Times 2 and/or 3 (presence or absence of a new reveal) from relevant baseline predictors. New reveals at Times 2 and 3 were also collapsed to have sufficient group size to examine predictors of new voluntary disclosures and involuntary discoveries. However, indirect disclosures could not be predicted over time due to insufficient group size. Difficulties with emotion regulation, depressive symptoms, loneliness, lifetime NSSI severity, self-efficacy to resist NSSI urges, and healthy coping at baseline were examined as predictors of new voluntary disclosures (yes = 68, no = 524) and involuntary discoveries (yes = 46, no = 546). Model fit for each model was examined, and predictors were removed based on their individual contribution to the model fit (significance of odds ratios) to create a best-fitting model. All statistical analyses were performed using IBM SPSS Statistics version 29 (IBM SPSS Statistics for Windows, 2024). Given that the analyses were largely exploratory, a liberal alpha of 0.05 was used.

### Missing Data

There was very minimal missing data within a wave (e.g., less than 1% at baseline) and participation retention was good (78.3% completed all three waves, 12.9% completed two waves, and 8.8% completed one wave). Given that some participants did not complete all three waves, reveals happening at Times 2 and 3 were coded as 1 for participants with who indicated one or more reveal, and 0 for all others, including those with missing data (which was minimal). This conservative re-coding approach was used to account for attrition and retain participants across time.

## Results

### Sample Characteristics

In the present sample, 72% of participants identified as women, 22% identified as men, 4% identified as nonbinary, and 2% reported they were unsure. From a multiple-selection checklist, 34.6% of participants identified as East Asian, 24.3% as White, 20.4% as South Asian, 7.8% as Arab/West Asian, 6.1% as Black, 5.2% as Hispanic, 5.1% as Filipino, 4.4% as South East Asian, 2.2% as West Indian. Additionally, 3.1% of participants specified other ethnic identities including bi-racial, Jewish, and South African.

### NSSI Behaviors and Functions

All participants in the present sample (N = 592) reported a lifetime history of NSSI engagement. At Times 1, 2, and 3, 57.1%, 40.9%, and 32.1% of participants reported recent NSSI engagement (past 3 months), respectively. At Times 1 and 2, the most common recent NSSI behaviours included banging or hitting self (52.5%; 40.7%), biting (43.8%; 38.7%), and severe scratching (41.9%; 36.1%). At Time 3, the most common recent NSSI behaviours included banging or hitting self (32.3%), severe scratching (31.1%), and biting (29.7%). Across all three time points, the most commonly endorsed functions of NSSI engagement included affect regulation, self-punishment, anti-dissociation, and marking distress.

### Prevalence of NSSI Reveals

Overall, 50% of participants reported experiencing an NSSI reveal at some point across their first year of university. At Time 1, 56.8% of participants reported having experienced a reveal in their lifetime and 43.1% of participants reported a recent (past 3 months) reveal experience. At Times 2 and 3, 16% and 11% of participants reported having experienced a recent (past 3 months) reveal, respectively. On average, participants had 2 different types of sources (e.g., friend and parent) come to know of their NSSI at each time point. At Time 1, participants reported 4 unique individuals came to know of their NSSI on average in the past 3 months (e.g., two friends and two parents), while at both Times 2 and 3, participants reported 2 unique individuals came to know of their NSSI on average in the past 3 months.

The prevalences of three specific reveal experiences were examined (see Figure 1). Overall, 74% of participants who experienced a reveal in their first year of university had at least one experience where they voluntarily disclosed their NSSI. At Times 1, 2, and 3, 54%, 57% and 49% of participants reported experiencing at least one voluntary disclosure, respectively.

**Figure 1.**
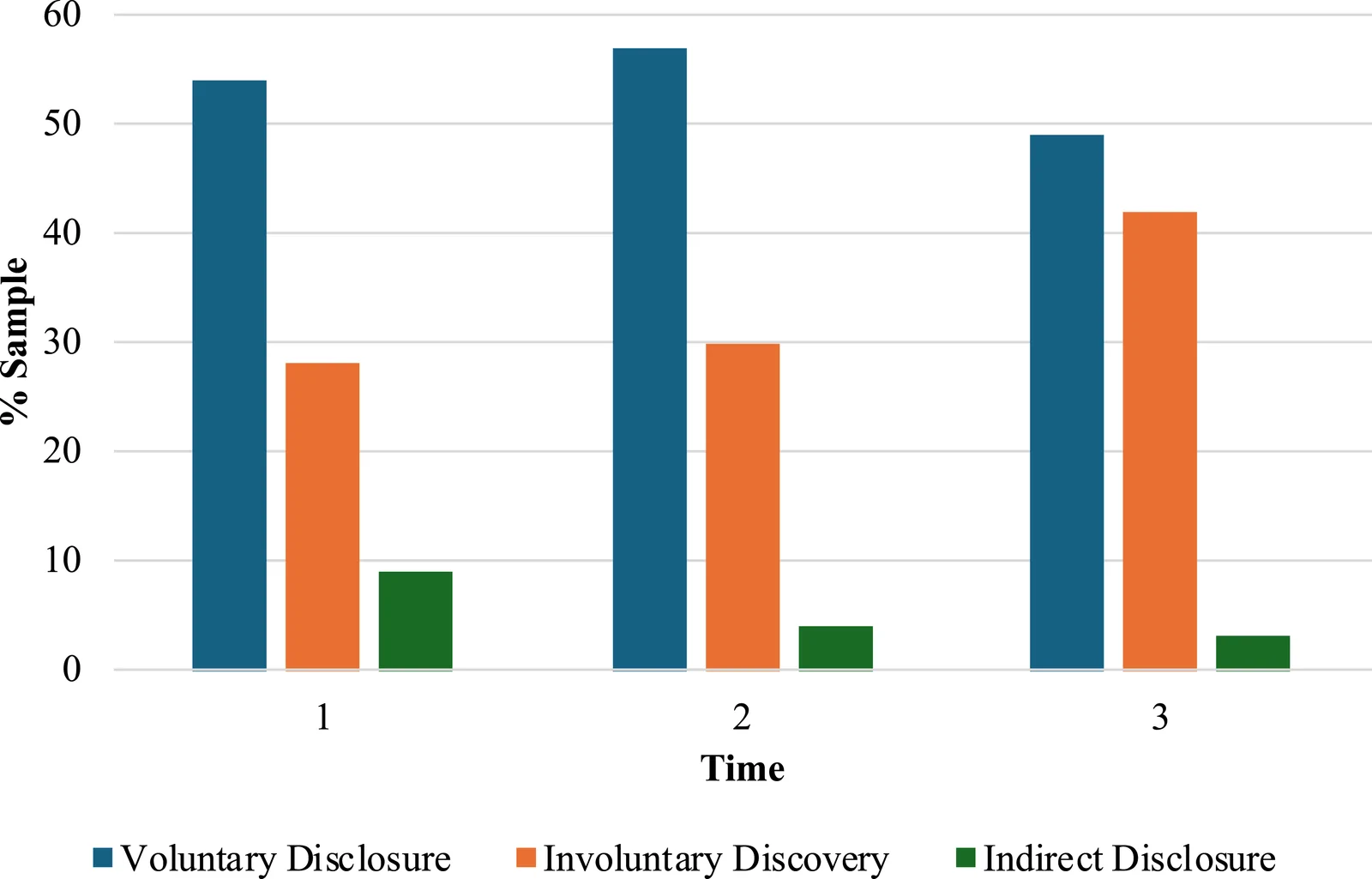
Prevalence of diverse NSSI reveal experiences across the first year of university.*Note.* Bars represent the number of students who reported having experienced at least one incidence of the specific reveal type within the past 3 months of a given time point. This sample included participants who indicated a lifetime history of NSSI and reported experiencing at least one NSSI reveal at some point across their first year of university

At Times 1, 2 and 3, 90%, 79%, and 84% of these students reported at least one voluntary disclosure to an informal source (e.g., family member, partner), while 28%, 30%, and 38% reported at least one voluntary disclosure to a formal source (e.g., doctor, mental health clinician), respectively. Furthermore, 61% of participants who experienced a reveal in their first year of university indicated having at least one involuntary discovery of their NSSI. At Times 1, 2, and 3, 28%, 30%, and 42% of participants reported at least one involuntary discovery, respectively. Finally, 5% of participants who experienced a reveal in their first year of university had at least one indirect disclosure of their NSSI. At Times 1, 2, and 3, 9%, 4% and 3% of participants reported at least one indirect disclosure, respectively.

### Primary Analyses

#### Voluntary Disclosures

A binary logistic regression was conducted, including difficulties with emotion regulation (DERS-SF), depressive symptoms (CESD-R), loneliness (ULS-8-SF), lifetime NSSI severity, self-efficacy to resist NSSI urges (SEASA), and healthy coping (CI) at baseline as predictors of voluntary disclosures over the first year of university (i.e., at Times 2 and 3; 0 = no voluntary disclosure, 1 = voluntary disclosure). A test of this full model versus a model with intercept only was significant, χ^2^ (6) = 12.92, *p* = .044; however, some predictors in this model did not improve model fit. As such, predictors including depressive symptoms and self-efficacy to resist NSSI urges were removed based on their individual contribution to the model fit (significance of odds ratios) to create a best-fitting model. A test of this model (including difficulties with emotion regulation, loneliness, lifetime NSSI severity, and healthy coping as predictors) versus a model with intercept only was significant, χ^2^ (4) = 10.02, *p* = .040. This model correctly classified 88.6% of cases (see Table 1 for regression coefficients, Wald statistics, odds ratios and 95% confidence intervals). Results indicated that students who reported greater lifetime NSSI severity were more likely to later experience a voluntary disclosure (see Figure 2). Difficulties with emotion regulation, loneliness, and healthy coping did not significantly predict later voluntary disclosures.

**Table 1.** Final Binary Logistic Regression Predicting Voluntary Disclosure of NSSI at Times 2 and 3. (N = 592, 68 yes, 524 no)

Predictor	*B*	Wald chi-square	Odds ratio (ExpB)	*p* value	95% confidence interval
Difficulties with emotion regulation (DERS-SF)	.372	3.096	1.451	.079	[0.96, 2.20]
Loneliness (ULS-8-SF)	−.252	1.220	0.777	.269	[0.50, 1.22]
Healthy coping (CI)	−.018	0.004	0.983	.947	[0.58, 1.65]
Lifetime NSSI severity	.175	4.446	1.191	.035	[1.01, 1.40]

**Figure 2.**
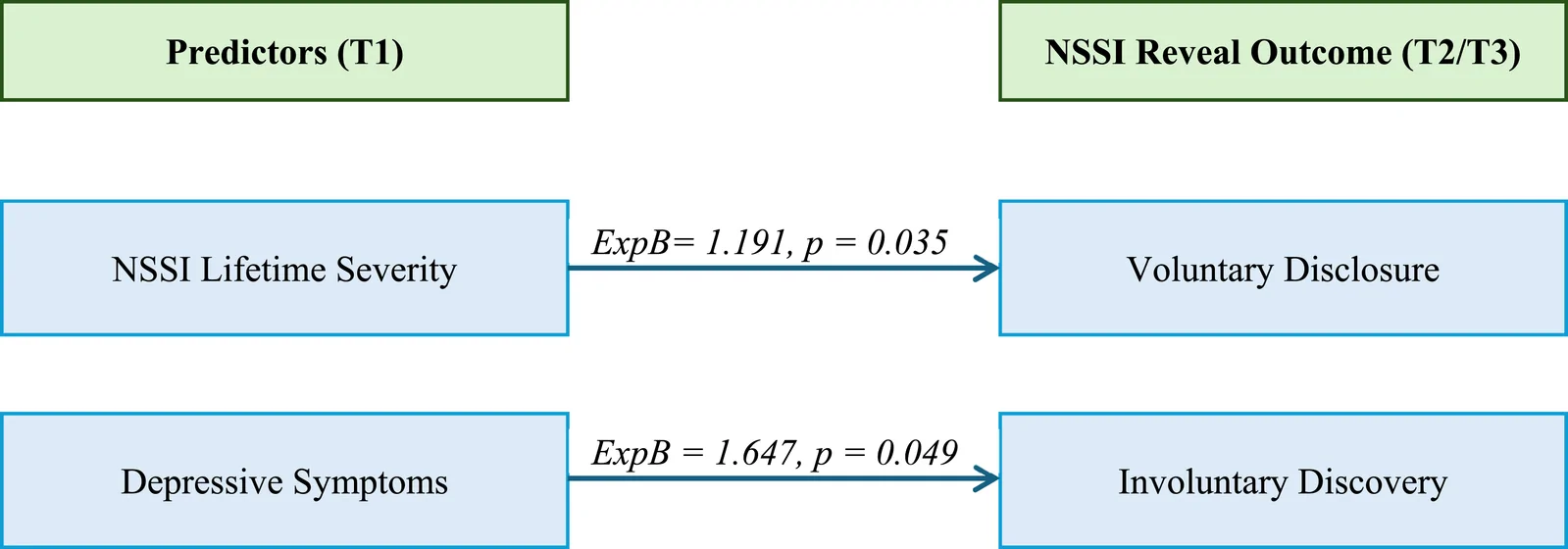
Binary logistic regression model results for significant predictors of NSSI reveal experiences across the first year of university

#### Involuntary Discoveries

A binary logistic regression was conducted, including difficulties with emotion regulation (DERS-SF), depressive symptoms (CESD-R) loneliness (ULS-8-SF), lifetime NSSI severity, self-efficacy to resist NSSI urges (SEASA), and healthy coping (CI) at baseline as predictors of involuntary discoveries over the first year of university (i.e., at Times 2 and 3; 0 = no involuntary discovery, 1 = involuntary discovery). A test of this full model versus a model with intercept only was statistically significant, χ^2^ (6) = 14.60, *p* = .024. This model correctly classified 92.2% of cases (see Table 2 for regression coefficients, Wald statistics, odds ratios, and 95% confidence intervals). Results indicated that students who reported greater depressive symptoms were more likely to later experience an involuntary discovery of their NSSI (see Figure 2). Furthermore, students reporting greater self-efficacy to resist NSSI urges were marginally less likely to later experience an involuntary discovery. Difficulties with emotion regulation, loneliness, lifetime NSSI severity, and healthy coping did not significantly predict later involuntary discoveries of NSSI.

**Table 2.** Binary Logistic Regression Predicting Involuntary Discovery of NSSI at Times 2 and 3. (N = 592, 46 yes, 546 no)

Predictor	*B*	Wald chi-square	Odds ratio (ExpB)	*p* value	95% confidence interval
Difficulties with emotion regulation (DERS-SF)	−.440	2.208	0.644	.137	[0.36, 1.15]
Loneliness (ULS-8-SF)	−.130	.220	0.878	.639	[0.51, 1.51]
Healthy coping (CI)	.295	.859	1.343	.354	[0.72, 2.51]
Self-efficacy to resist NSSI urges (SEASA)	−.133	3.727	0.875	.054	[0.76, 1.00]
Lifetime NSSI severity	.175	3.169	1.192	.075	[0.98, 1.45]
Depressive symptoms (CESD-R)	.499	3.862	1.647	.049	[1.00, 2.71]

## Discussion

Despite the post-secondary years representing a key period of increased vulnerability for NSSI (Kiekens et al., 2023), little is known about NSSI reveals in the post-secondary context. Existing work has often conflated the different ways in which NSSI may be revealed to others, and limited longitudinal work impedes an understanding of what may promote these experiences in the post-secondary context over time. To address these gaps, multiple relevant predictors of different NSSI reveal experiences were examined (i.e., voluntary disclosures, involuntary discoveries, and indirect disclosures) across the first year of university using a longitudinal design. As expected, lifetime NSSI severity was relevant to voluntary disclosures. Additionally, a novel finding was that depressive symptoms and self-efficacy to resist NSSI urges were relevant to involuntary discoveries.

NSSI reveals were highly prevalent among students with a lifetime history of NSSI, with 50% of these students reporting a reveal experience at some point across their first year of university. This finding adds to emerging literature that highlights the transition to university as a key period where NSSI experiences are shared (e.g., Ammerman & McCloskey, 2021; Mirichlis et al., 2022; Simone & Hamza, 2023). Although the transition to university represents a time of vulnerability for NSSI engagement, it may also represent a window of opportunity for students to receive social support in a new context, providing a potential gateway to help-seeking and access to university mental-health resources (Kim et al., 2025). Voluntary disclosures were the most prevalent type of reveal that students reported experiencing at some point across the year (74%), but involuntary discoveries were common as well (61%). These findings underscore the importance of disentangling different types of reveals to better understand how others come to know about students’ NSSI engagement in the post-secondary context.

### Predictors of Voluntary Disclosures

Binary logistic regression models showed that students reporting greater NSSI lifetime severity at baseline were more likely to later experience a voluntary disclosure. This finding is in line with previous work (Hasking et al., 2015; Simone & Hamza, 2021; Turner et al., 2016), which suggests that disclosure may be used as a means of signalling distress or help-seeking. This area of research has also indicated that individuals who engage in more severe NSSI behaviours may be at a greater risk of suicide (Anestis et al., 2015; Turner et al., 2013). Taken together, students who voluntarily disclose their NSSI appear to represent a higher risk population, underscoring why effective NSSI response and support are critical in the post-secondary context.

In this multivariate model, several potential predictors implicated in prior research did not significantly predict later voluntary disclosures. Much of prior work on disclosures has been cross-sectional (Simone & Hamza, 2020), so it is possible that it is more challenging to identify predictors of disclosures over time (e.g., 3 or 6 months later). Prior work has also not well differentiated voluntary disclosures and involuntary discoveries of NSSI, which may also help account for our discrepant findings. For example, both depressive symptoms and self-efficacy to resist NSSI urges were found to be relevant to involuntary discoveries across time, but did not emerge as predictors of voluntary disclosures in the present study. Thus, some of the predictors previously implicated in disclosures may be more relevant to involuntary reveal experiences. It is also possible that some of the predictors studied do not well differentiate individuals who disclose from those who do not disclose during the early transition to university. Given that students were assessed as they transitioned into university, they may have been experiencing separation from previous socials supports and increased distress during this time. In the present study, most students reported feeling lonely (mean of 3/4 on the ULS-8-SF) and experienced moderate emotion dysregulation (mean 3/5 on the DERS-SF) at baseline. It may therefore be difficult to use these predictors to delineate students who did and did not disclose in the first year of university.

### Predictors of Involuntary Discoveries

Students who reported greater depressive symptoms at baseline were more likely to later experience an involuntary discovery of their NSSI. The current study is the first, to our knowledge, that has examined the impact of depressive symptoms on involuntary discoveries over time. The present findings suggest that depressive symptoms may be specifically relevant to involuntary rather than voluntary reveal experiences across time. It is possible that certain aspects of depressive symptoms (e.g., low motivation, apathy) may facilitate involuntary discoveries due to unconcealed visible signs of NSSI, but more research is required to understand mechanisms underlying the relation between depression and involuntary discoveries.

Although students’ self-efficacy to resist NSSI urges was not related to voluntary disclosures, it was found to be relevant to involuntary discoveries. Specifically, students with greater self-efficacy to resist NSSI urges at baseline were less likely, albeit marginally, to later report an involuntary discovery. One possible explanation is that the ability to resist NSSI urges results in reduced NSSI engagement and visible signs of self-injury. This may reduce the likelihood that students are asked about their lived experience with NSSI (i.e., involuntary discoveries). Interestingly, self-efficacy to resist NSSI urges has been conceptualized as a marker of NSSI recovery (Lewis & Hasking, 2021), but more research is required to understand the nature of NSSI reveal experiences that uniquely occur within the context of NSSI recovery.

### Implications

Findings from the present study hold important implications for university intervention efforts. Ensuring that students have access to resources to navigate NSSI reveal experiences on university campuses, and providing supports for coping with potential negative or stigmatizing responses to reveals is critical. Equipping students with knowledge regarding NSSI and strategies to respond effectively to reveals could further improve these experiences for both parties, reduce stigmatization, and foster understanding among peers. Additionally, training university staff on campuses (e.g., residence staff and student wellness program teams) in best practices for responding to NSSI reveals could play a pivotal role. These efforts may create a more supportive campus environment for students with lived histories of NSSI. Furthermore, increasing access to safe spaces where students can disclose their NSSI to mental health professionals skilled in providing therapeutic interventions is critical to promoting NSSI recovery on post-secondary campuses.

Increasing the accessibility of these services may be particularly vital for promoting the initiation of disclosure and help-seeking among students, particularly given present findings that post-secondary students were more likely to disclose to informal rather than formal supports, consistent with prior literature (Ammerman et al., 2021; Simone & Hamza, 2020). Furthermore, recent work has highlighted that involuntary discoveries are often characterized by reduced control (e.g., when and where the reveal occurs, and to whom the (Lewis & Hasking, 2023; Lewis & Hasking, 2023). As such, providing students with resources and strategies to cope with these unique reveal experiences could represent another important target for university intervention efforts.

### Limitations & Future Directions

The present study includes many strengths, including its large sample size, longitudinal design, and ability to disentangle reveal experiences that have been largely conflated in existing literature. However, the present study is not without limitations. First, the present study relied on retrospective self-reports, and may be subject to recall errors. Future studies could use shorter assessment intervals (e.g., monthly) or make use of experience sampling techniques (e.g., daily sampling) to mitigate recall errors. However, it’s important to note that disclosures occur infrequently, so researchers must balance sampling with likelihood of capturing new disclosure experiences. Second, although our large sample was representative of the university at which the present data was collected, the majority of participants identified as female. As such, the present findings may not represent the NSSI experiences of first year students of other gender identities. Future studies should investigate NSSI reveal experiences across the first year of university using a more gender diverse sample to increase generalization of findings. In line with this, the current study did not have enough power to examine differences across ethnicities and genders due to small sample sizes within certain subgroups. Future research should investigate how cultural and gender-related factors influence NSSI reveal experiences, as these contexts may be important for understanding stigma and other barriers to disclosure and help-seeking. Third, more work is required to understand the outcomes of different NSSI reveal experiences on student well-being, recovery, and help-seeking. Given the exploratory nature of this work, qualitative methods (e.g., thematic analysis) may be advantageous in obtaining rich insights to how NSSI reveal experiences occur in the post-secondary context (e.g., the role of depressive symptoms in involuntary discoveries, and impacts of these unexpected experiences in high-risk samples). Further, future research should also examine the modality of NSSI disclosure (e.g., online versus in-person), as online contexts could serve as an initial, lower-risk entry point in the disclosure process that may shape subsequent help-seeking trajectories. Finally, the present study was unable to predict indirect disclosures due to a small number of students reporting this experience. Future qualitative work could further help to elucidate factors involved in this unique reveal experience (e.g., quality of relationships, greater concerns from confidants, more severe NSSI behaviours). In addition, it is important to note that the present study was not pre-registered, and this work should be considered exploratory. Findings should therefore be interpreted with caution until replicated in future research.

## Conclusion

The present study aimed to examine multiple relevant predictors of different NSSI reveal experiences across the first year of university using a longitudinal design. Findings provide critical insights to the nuanced experiences of NSSI reveals during the transition to university, a period marked by heightened vulnerability but also significant opportunities for intervention. Indeed, NSSI was often revealed in this context, with half of students with a lifetime history of NSSI reporting an NSSI reveal at some point across the first year of university. Findings indicated that students reporting greater NSSI lifetime severity at baseline were more likely to later experience a voluntary disclosure, and students reporting greater depressive symptoms at baseline were more likely to later experience an involuntary discovery of their NSSI. This work highlights the importance of understanding the diverse pathways through which these distinct experiences occur and their unique implications for student well-being. The present findings further underscore the need for targeted interventions that equip students with resources and knowledge to navigate these experiences and promote supportive responses from peers and university staff. Future research should continue to explore the complexities of NSSI reveals, including longitudinal trajectories and qualitative dimensions, to inform comprehensive strategies for supporting post-secondary students with lived experiences of NSSI.
